# BH3-only proteins: the thorny end of the ER stress response

**DOI:** 10.1038/cddis.2017.283

**Published:** 2017-06-22

**Authors:** Jason A Glab, Marcel Doerflinger, Hamsa Puthalakath

**Affiliations:** 1 Department of Biochemistry & Genetics, La Trobe Institute for Molecular Science, La Trobe University, Kingsbury Drive, Bundoora, Victoria 3086, Australia

The endoplasmic reticulum (ER) is the hub of protein trafficking inside the cell. Secreted, membrane-bound and organelle-targeted proteins are all trafficked through the ER before being post-translationally modified (i.e. glycosylated and folded), making them competent for exporting. This involves coordinated functioning of chaperones, glycosyltransferases and protein disulfide isomerases. Perturbation of ER homeostasis—such as disturbance of ATP, calcium levels or change in the redox status—can affect protein folding and lead to protein aggregation and ER stress. ER stress leads to two divergent cellular responses: the unfolded protein response—where cells mitigate the stress by reducing protein load and upregulating the production of chaperones; should the cells be overwhelmed by the stress, they eventually reach a point of no return, at which point they instigate apoptosis. While the former response is necessary for developmental processes—such as plasma and dendritic cell differentiation and muscle fiber formation^1^— apoptosis is often harmful. It is attributed to many human pathologies, such as cystic fibrosis, *α*1-antitrypsin deficiency, thyroglobulin deficiency and diabetes insipidus.^1^ For this reason, the apoptotic response of ER stress has been the subject of intense study for the past couple of decades.

Understanding the molecular mechanism of ER stress-induced apoptosis has been a theorist’s paradise, and has been highly controversial. One of the earliest reports on the mechanism was by Nakagawa *et al.*,^2^ who claimed that caspase-12 was the main instigator of ER stress-mediated apoptosis. Caspase-12 appeared to be localized to the ER and activated by ER stress, and genetic ablation of caspase-12 in mice resulted in protection of cortical neurons from *β*-amyloid-induced apoptosis. However, subsequent study by Saleh *et al.*^3^ contradicted this on a number of levels. First, *caspase-12*^*−/−*^ mouse embryonic fibroblasts (MEFs) did not offer protection against apoptosis induced by a variety of ER stress inducing agents. In addition, apart from a subgroup of the African population, most human populations do not produce functional caspase-12.^4^ The role of caspase-12 in mice and in human populations, where the functional protein is expressed, appears to be in modulating inflammatory response to infections. Lack of functional caspase-12 in humans prompted Hitomi *et al.*^5^ to suggest that caspase-4 was the equivalent of caspase-12 in humans and had a crucial role to play in ER stress-induced apoptosis in human cells in response to β-amyloids. However, this assertion was also discredited in subsequent studies,^6^ where both caspase-12 and caspase-4 were found not to be involved in ER stress-induced apoptosis. In cells expressing either murine caspase-12 or human caspase-4, ER stress-induced apoptosis could be blocked by over expression of Bcl-XL or by a dominant negative form of caspase-9—suggesting a role for the mitochondrial pathway.

There is mounting evidence for the role of BH3-only members of the Bcl-2 family in this process. While earlier studies defined a role for PUMA and NOXA in ER stress-induced apoptosis in neuronal cells and in MEFs,^7, 8, 9^ we had reported a role for BIM in lymphocytes, macrophages and epithelial cells.^10^ We also recently reported a role for ER stress-mediated BIM induction in lymphocytes during sepsis in mice.^11^ Thus, there appears to be a division of labor among various BH3-only proteins (i.e. PUMA largely in neuronal cells, NOXA and PUMA in fibroblasts and BIM in lymphocytes, myeloid cells as well as epithelial cells in inducing ER stress-mediated apoptosis). There was a suggestion that the BH3-only proteins BIM and PUMA were the upstream modulators of ER stress—where these two proteins could interact with IRE1*α* and regulate XBP-1 splicing, lymphocyte maturation and IgM secretion.^12^ However, our attempts to reproduce these results were unsuccessful.^13^ Our recent results,^14^ using a variety of gene knockout cell lines and via activated caspase-specific pull-down experiments, unequivocally show a central role for the mitochondrial pathway and BH3-only proteins in this apoptotic process. Again, the role of individual BH3-only protein appears to be cell type-specific (Figure 1).

Elucidation of the precise molecular mechanism(s) of ER stress-induced apoptosis is crucial for developing therapeutics for many diseases, particularly for cancer. Hypoxic conditions in the microenvironment and increased demand for protein synthesis mean tumor cells have high levels of ER stress and evolve mechanisms to overcome the resulting apoptosis. For this reason, a great many chemotherapeutic drugs—including NSAIDs, proteasomal inhibitors and HDAC inhibitors—all work by inducing the apoptotic arm of the ER stress pathway.^15^ Finally, establishing the role of BH3-only proteins in ER stress-induced apoptosis is also crucial in evaluating the potential benefits of new generation cancer therapeutics based on ‘BH3 mimetics’.

## Figures and Tables

**Figure 1:**
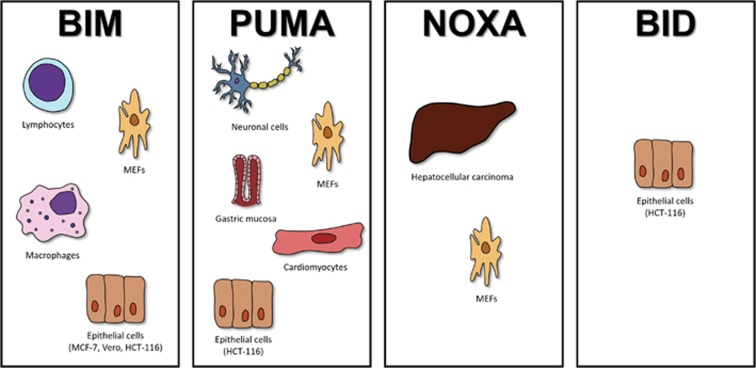
BH3-only proteins and the division of labor. The apoptotic response of ER stress is largely controlled by the BH3-only proteins; however, their role is cell/tissue-specific
